# Antisocial cognition as a mediator of the peer influence effect and peer selection effect in antisocial adolescents

**DOI:** 10.1007/s00787-020-01695-1

**Published:** 2020-12-16

**Authors:** Ashley-John Brewer, Rob Saunders, Pasco Fearon, Peter Fonagy, David Cottrell, Abdullah Kraam, Stephen Pilling, Elizabeth Simes, Alisa Anokhina, Stephen Butler

**Affiliations:** 1 Research Department of Clinical, Educational and Health Psychology, University College London, London, UK; 2 Centre for Outcomes Research and Effectiveness, Research Department of Clinical, Educational and Health Psychology, University College London, London, UK; 3 Leeds Institute of Health Sciences, University of Leeds, Leeds, UK; 4 University of Leeds and Rotherham Doncaster and South Humber NHS Foundation Trust, Leeds, UK

**Keywords:** Mediation analysis, Antisocial behaviour, Adolescence

## Abstract

The peer influence and peer selection effects are two widely replicated findings in the criminological literature that refer to the predictive relationship between antisocial behaviour and delinquent peer association as well as between delinquent peer association and antisocial behaviour, respectively. Research suggests that antisocial cognition might constitute a causal mechanism underlying part of these effects. This study investigated the extent that the peer influence and peer selection effects are mediated by one key aspect of antisocial cognition—*beliefs and attitudes supporting peer conflict*. This study examined whether beliefs and attitudes supporting peer conflict mediated the relationship between delinquent peer association and volume of self-reported antisocial behaviour and vice-versa, across a 1-year follow-up period, in 683 (433 male, 250 female) British adolescents (mean age: 13.8 years) with a history of serious antisocial behaviour. Participants completed measures at baseline and 6, 12 and 18 months thereafter. Findings indicated that beliefs and attitudes supporting peer conflict partially mediated the peer influence and peer selection effects, explaining a substantial proportion of the total effect in the peer influence (i.e., 26%) and peer selection (i.e., 17%) models. These results suggest that beliefs and attitudes supporting peer conflict could explain part of the mechanism underlying the peer influence and peer selection effects in adolescents with a history of serious antisocial behaviour.

**Supplementary Information:**

The online version contains supplementary material available at 10.1007/s00787-020-01695-1.

## Introduction

Serious and persistent antisocial behaviour during adolescence can have significant and costly long-term outcomes for individuals, their families, and society [1, 2]. From a developmental perspective, peer factors come to dominate during adolescence and young people’s associations with delinquent peers is a well-established risk factor for youth antisocial behaviour [3, 4]. Risk-focused research in the last 30 years has consistently demonstrated a predictive relationship between delinquent peer association and antisocial behaviour and between antisocial behaviour and delinquent peer association [5–9]. The former of these relationships is commonly known as the *peer influence effect* and the latter the *peer selection effect.*

The peer influence effect is believed to involve adolescents adjusting their behaviours, attitudes and beliefs to conform to those of their friends. Dishion and colleagues have developed the construct of ‘deviancy training’, where communication and interactions between deviant peers reinforce changes toward antisocial behaviours through gestures, talk and behaviour [10, 11]. The peer selection effect, on the other hand, occurs when young people actively look for peers that tend to match their own behaviours, attitudes and beliefs. While the importance of peer influence and peer selection effects are well established, much less is known about why youth would select or be influenced by certain peers. Understanding how influence and peer selection effects may result in antisocial behaviour, and how engagement in antisocial acts may bring about peer influence and peer selection effects would improve understanding of the proximal mechanisms underlying the development and persistence of antisocial behaviour and highlight potential targets for intervention.

Research suggests that the peer influence and peer selection effects could be transmitted by similar causal mechanisms, namely certain forms of *antisocial cognition* [12–15]. Antisocial cognition has no formal definition, but has been used to refer to attitudes, beliefs, and thinking supportive of crime [16]. Congruent with this definition, the role of antisocial cognitions as a mediator between peer influence and peer selection effects and antisocial behaviour is suggested by numerous theories of criminal conduct [17–19] and recent empirical studies [12–15]. For example, Sutherland [18] suggested that attitudes favourable to law-breaking behaviour learned in the context of deviant peer associations were the primary mechanisms for the transmission of peer delinquency, while Akers [17] identified both antisocial attitudes and modelled deviant behaviour as coming together to influence deviant peer associations and delinquent behaviour. More recently, in a series of empirical studies [12–15], Walters examined the extent to which antisocial cognitions mediated the peer influence and peer selection effects. In an initial mediation analysis [12], Walters assessed *proactive criminal thinking* using four items asking about the acceptability of stealing in given situations and discovered that this measure partially mediated the peer influence effect in youths aged 10–18 years, without a history of serious offending, from the British Offending, Crime and Justice Survey. Two successive studies found that proactive criminal thinking regarding the acceptability of violence, breaking rules and lying in certain situations, and *attitudes toward deviance*, independently and conjointly mediated part of the peer influence effect in a sample of American youths aged 11–17 years from the National Youth Survey [14, 15]. Finally, in a group of male adjudicated adolescents aged 14–19 years from the Pathways to Desistance study [13], several measures of antisocial cognition partially mediated the peer influence effect and peer selection effect, respectively. These theoretical and empirical findings suggest that aspects of antisocial cognitions such as antisocial beliefs and attitudes could explain at least part of the peer influence and peer selection effects.

Empirical research has shown that antisocial beliefs and attitudes are associated with antisocial behaviour in cross-sectional and longitudinal studies of high school students and young offenders [20–25]. In a sample of youths aged 10–18 years without a history of serious offending from the British Offending, Crime and Justice Survey, an initial mediation analysis suggested that beliefs supportive of stealing partially mediated the peer influence effect [12]. Subsequent studies found that beliefs and attitudes supportive of aggressive and non-aggressive antisocial behaviours independently and conjointly mediated part of the peer influence effect in a sample of American youths aged 11–17 years from the National Youth Survey [14, 15]. These findings suggest that antisocial beliefs and attitudes may partly account for the peer influence and peer selection effects [12–15].

While these findings are encouraging and serve to establish links between antisocial beliefs and attitudes and these deviant peer processes, they tend to employ rather broad indices of antisocial cognitions with limited reliability and validity. Furthermore, we know of only one study that has examined aspects of antisocial cognition as a mediator of the peer influence and selection effects in young people with a history of serious antisocial behaviour [13], yet this group is the most likely to offend across the lifespan [26–28]. Our approach is to aim for greater specificity by identifying specific domains of antisocial beliefs and attitudes, and in doing so examine cognitions that are linked to certain types of peer interactions. Consequently, we identify antisocial beliefs and attitudes supportive of peer conflict as a factor that may specifically contribute to both peer influence and peer selection effects. From this perspective, we propose that young people with beliefs and attitudes accepting of engaging in conflict with peers, physical fighting, and aggressive peer interactions, are more likely to be influenced by like-minded antisocial peers, and to gravitate toward each other.

There are emerging findings that highlight the importance of beliefs and attitudes supportive of peer conflict for the development of antisocial behaviour. In two initial studies of the psychometric properties of the Antisocial Beliefs and Attitudes Scales (ABAS), a peer conflict factor demonstrated good reliability and validity and predicted self-reported antisocial behaviour in primary and secondary school children in Canada [29] and the U.K. [20], as well as self- and parent-reported antisocial behaviour in British young offenders [20]. These findings suggest that young people who endorse beliefs and attitudes supporting peer conflict are more likely to engage in antisocial behaviour and that the presence of such beliefs and attitudes may be fundamental to understanding the development and persistence of antisocial behaviour. The predictive relationships observed by Butler and colleagues [20, 29] and the fact that delinquent beliefs have been shown to positively correlate with both delinquent behaviour and delinquent peer association [30], indicate that beliefs and attitudes supporting peer conflict could explain part of the peer influence and peer selection effects. Finally, in an interesting and relevant meta-analysis of 20 studies, adherence to subcultural “street code” beliefs had a positive effected on youth offending and in particular, violent offending [31]. Street code beliefs involved treating young offenders with the deference and respect, earned amongst their peers by showing that they are not to be trifled or messed with, that they are ready to display threatening behaviour, and that they will defend themselves against instances of peer conflict such as disrespect or interpersonal aggression. This meta-analysis suggests that antisocial beliefs and attitudes toward peer conflict are robustly associated with youth offending and may be central to understanding the peer ecologies that promote youth criminogenic behaviour in urban communities.

In summary, affiliating with delinquent peers who endorse beliefs and attitudes supporting peer conflict could provide an environment that fosters and reinforces such antisocial cognitions. The existence and strengthening of these cognitions might then increase the likelihood of conflict with peers or other forms of antisocial behaviour, and once a person has engaged in antisocial behaviour, they might be more likely to associate with peers who have engaged in similar behaviours. As reported above, this view is consistent with several eminent and empirically supported theories of crime [17, 18, 30, 32, 33] and accumulating empirical evidence.

The current study investigates whether beliefs and attitudes supporting peer conflict mediate the peer influence and peer selection effects in adolescents with a history of serious behaviour. This was explored in two mediation analyses of longitudinal data collected from adolescents with a history of serious antisocial behaviour over an 18-month period. It was hypothesised that: (1) delinquent peer association would predict beliefs and attitudes supporting peer conflict, which in turn would predict self-reported antisocial behaviour and, thus, beliefs and attitudes supporting peer conflict would mediate the peer influence effect; and (2) that self-reported antisocial behaviour would predict beliefs and attitudes supporting peer conflict, which in turn would predict delinquent peer association and, thus, beliefs and attitudes supporting peer conflict would mediate the peer selection effect.

## Method

### Design and participants

This paper reports a secondary analysis of data from the Systemic Therapy for at Risk Teens (START) study (trial registration ISRCTN77132214); a national multicentre pragmatic clinical randomised controlled trial, comparing the efficacy of Multisystemic therapy (MST) with management as usual (MAU) in reducing risk of out-of-home placement in adolescents due to significant antisocial behaviour. Young people with moderate-to-severe antisocial behaviour were recruited from social services, youth offending teams, schools, child and adolescent mental health services (CAMHS), and voluntary services, with full details on methodology and participant eligibility available from the START study protocol and report [34, 35]. For the current analysis, data were used from all 683 (63.4% male, 36.6% female) participants aged 11–17 years (mean = 13.81) who entered the START trial. Most participants were White British/European (78%), and from lower income households (62%). No further inclusion criteria were applied for the current analysis, and descriptive statistics for the sample are presented in Table 1. Consenting participants completed a battery of questionnaires at baseline, after which approximately half were randomised to MST or MAU. MST was delivered over 3–6 months (average duration 139 days). Follow-up assessments were conducted six, 12 and 18 months after baseline and participants were not contacted between assessments. The study thus comprised four data collection timepoints.

**Table 1:** Descriptive statistics for study variables

Variable	*N*	*M*	SD
Age	683	13.81	1.41
PC-baseline	683	6.77	4.01
PC-12	486	6.52	3.58
DPA-baseline	683	4.95	4.65
DPA-6	672	4.76	4.18
DPA-18	461	4.58	4.19
Vol-baseline	683	19.59	17.06
Vol-6	672	15.96	14.42
Vol-18	461	8.74	8.28

Variable	*N*	%
Gender		
Male	433	63.4
Female	250	36.6
Treatment		
MST	342	50.1
MAU	341	49.9
Ethnicity		
White British/European	535	78.3
Black African/Caribbean	71	10.4
Asian	16	2.3
Mixed/Other	51	7.5
Unknown	10	1.5
Socio-economic status		
Low	424	62
Medium	178	26
High	68	10
Unknown	13	2

* N* number of non-missing cases, *M* mean; *SD* standard deviation, % percentage, *Age* age at study entry, *PC-baseline* beliefs and attitudes supporting peer conflict at baseline, *PC-12* beliefs and attitudes supporting peer conflict 12 months after randomisation, *DPA-baseline* delinquent peer association at baseline, *DPA-6* delinquent peer association 6 months after randomisation, *DPA-18* delinquent peer association 18 months after randomisation, *Vol-baseline* volume of antisocial behaviour at baseline, *Vol-6* volume of antisocial behaviour 6 months after randomisation, *Vol-18* volume of antisocial behaviour 18 months after randomisation, *Treatment* treatment group, *MST* multisystemic therapy, *MAU* management as usual, Socio-economic status: *low* ross household income < £15,000 per year, *medium* Gross household income £15,001 to £30,000 per year, *high* Gross household income > £30,000 per year

### Measures

#### Delinquent peer association

Delinquent peer association was measured using the ‘Your Friends’ subscale of the Self-Report Delinquency measure (SRD [36]), which comprises seven items asking about respondents’ peer involvement in antisocial behaviour during the last 6 months (e.g., substance use, truancy, theft, violent behaviour). Items are rated on Likert scales, which vary between items, and individual item scores are summed to form a total score for delinquent peer association in the last 6 months (range 0–20). The subscale has demonstrated split-half reliability [36] and construct validity in relation to self-reported delinquency in early-to-mid adolescence [36, 37]. Excellent reliability (Cronbach’s alpha = 0.92) was reported in the study sample averaged across the included timepoints [34].

#### Antisocial behaviour

Antisocial behaviour was measured using the Volume of Delinquency subscale of the SRD [36] which comprises 21 items about respondents’ involvement in antisocial behaviour during the last six months. Items are rated on Likert scales, which vary between items, and individual item scores are summed to form a total score for volume of antisocial behaviour in the last 6 months (range 0–153). The subscale has demonstrated split-half reliability [36, 37] and concurrent validity in relation to officially recorded delinquency in early-to-mid adolescence [37].

#### Antisocial beliefs and attitudes

Beliefs and attitudes supporting peer conflict were measured using the peer conflict factor of the ABAS [20, 29], which comprises 10 items asking about respondents’ beliefs and attitudes about peer conflict (e.g., *Fighting is cool when you’re with a group of kids, It’s ok to walk away from a fight, It’s fun and exciting to belong to a gang*). Items are rated on a three-point Likert scale and summed to form a total score for beliefs and attitudes supporting peer conflict (range 0–20). The peer conflict factor has demonstrated adequate internal consistency (Cronbach’s alpha = 0.77) and test–retest reliability (*r* = 0.77) over an 8-week period [20], as well as concurrent, predictive, and construct validity in community and offending samples of adolescents [20, 29]. Excellent reliability (Cronbach’s alpha = 0.93) was reported in the study sample averaged across the included timepoints [34].

### Statistical analysis

Mediation analysis was performed in Stata 14 [38]. Information on mediation analysis is presented in Supplementary Materials A. Age, gender, socioeconomic status and treatment group (MST/MAU) did not correlate with any variables in this study and were therefore excluded from the main mediation analyses.

Two mediation models were specified. The peer influence model tested whether antisocial beliefs and attitudes supporting peer conflict 12 months after randomisation mediated the relationship between delinquent peer association 6 months after randomisation and volume of self-reported antisocial behaviour 18 months after randomisation. The peer selection model examined whether antisocial beliefs and attitudes supporting peer conflict 12 months after randomisation mediated the relationship between volume of self-reported antisocial behaviour 6 months after randomisation and delinquent peer association 18 months after randomisation. Models were specified from the 6-month time point and therefore after the MST intervention had been received (MST was delivered in the first 3–6 months of the trial). Baseline precursor measures of mediator and dependent variables were included in mediation models and allowed to covary to control for the effects of these measures on these variables.

Specified models were recursive, over identified and estimated using the Satorra–Bentler estimator in Stata. Sensitivity testing was conducted using Kenny’s [39] “failsafe ef” procedure to understand how much an unobserved covariate would need to correlate with mediator and dependent variables to reduce the relationship between these variables to zero and, thus, confound any observed indirect effect. The robustness of the indirect effects were further explored by calculating bias-corrected bootstrapped confidence intervals (5000 bootstrap replications), and controlling for START trial intervention received as well as age and gender in additional sensitivity analyses. Further sensitivity analyses were conducted controlling for the timepoint 6-months before included variables (i.e. the 6-month peer conflict score, as well as the 12-month volume of self-reported antisocial behaviour and 12-month delinquent peer association for the peer influence and peer selection models, respectively) to additionally control for the potential impact of START trial intervention.

There was no evidence of multicollinearity between the control and predictor variables for either model estimated (peer influence model: tolerance = 0.732–0.971, VIF = 1.030–1.366; peer selection model: tolerance = 0.769–0.977, VIF = 1.024–1.301).

### Missing data

Complete data for the 13 variables in this study was available for 370 participants (54.2%). Eight variables had less than two percent missing data and the remaining five variables had between 19 and 33% missing data. Variables with over 30% missingness included delinquent peer association (32.5%) and volume of self-reported antisocial behaviour (31.5%) 18 months after randomisation.

Little’s Missing Completely at Random test showed that the pattern of missing data was *missing completely at random*, *X*^*2*^ (194, *N* = 461–683) = 214.39, *p* = 0.15. Missing data were, therefore, handled using expectation maximisation as implemented in SPSS.

## Results

### Peer influence effect

**Fig. 1:**
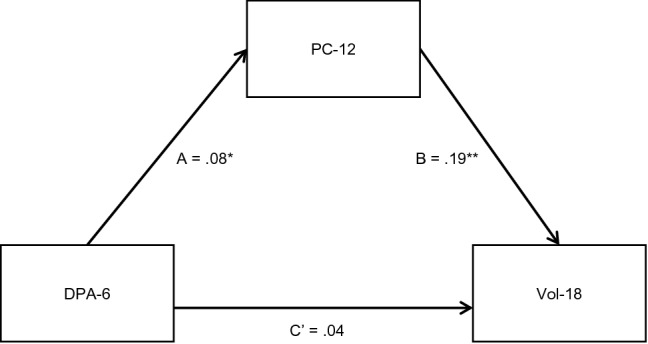
Path diagram for the peer influence mediation model. *N* = 663. Standardised beta coefficients are reported and precursor variables are not shown; *p < .05, **p < .01. *DPA-6* delinquent peer association 6 months after randomisation, *PC-12* beliefs and attitudes supporting peer conflict 12 months after randomisation, *Vol-18* volume of antisocial behaviour 18 months after randomisation

Pairwise correlations between the variables specified in the peer influence model are presented in the supplementary materials Table B1. A mediation analysis was performed to examine whether beliefs and attitudes supporting peer conflict 12 months after randomisation mediated the relationship between delinquent peer association six months after randomisation and volume of self-reported antisocial behaviour 18 months after randomisation. Findings are presented in Table 2 and Fig. 1.

**Table 2:** Peer influence effect: direct, total and indirect effects

	Direct effects				
Path	*B* (95% CI)	*SE B*	β	*z*	*p*
DPA-6 to PC-12 (Path A)	0.07(0.006;0.132)	0.03	0.08	2.13	0.03
PC-12 to Vol-18 (Path B)	0.44(0.261;0.614)	0.09	0.19	4.86	< 0.001
DPA-6 to Vol-18 (Path C’)	0.09(-0.074;0.247)	0.08	0.04	1.06	0.29
PC-baseline to PC-12	0.39(0.323;0.452)	0.03	0.43	11.83	< 0.001
Vol-baseline to Vol-18	0.14(0.105;0.179)	0.02	0.29	7.55	< 0.001

Total and indirect effects					
DPA-6 to Vol-18	*B* (95% CI)	*SE B*	β	*z*	*p*
Total effect	0.12(-0.044;0.278)	0.08	0.06	1.42	0.15
Indirect effect	0.03(0.001;0.059)	0.15	0.02	2.02	0.04

*B (95% CI)* unstandardized coefficient with 95% confidence intervals, *β* standardised coefficient, *z* asymptotic z-test, *p* statistical significance level of the asymptotic z-test; *N* 663. *PC-baseline* beliefs and attitudes supporting peer conflict at baseline, *PC-12* beliefs and attitudes supporting peer conflict 12 months after randomisation, *DPA-6* delinquent peer association 6 months after randomisation, *Vol-baseline* volume of antisocial behaviour at baseline, *Vol-18* volume of antisocial behaviour 18 months after randomisation

Delinquent peer association 6 months after randomisation had a significant direct effect on beliefs and attitudes supporting peer conflict 12 months after randomisation (Path A) but did not have a significant direct effect on volume of self-reported antisocial behaviour 18 months after randomisation (Path C’), when controlling for the effect of beliefs and attitudes and volume of self-reported antisocial behaviour at baseline. Beliefs and attitudes supporting peer conflict 12 months after randomisation had a significant direct effect on volume of self-reported antisocial behaviour 18 months after randomisation (Path B), when controlling for beliefs and attitudes supporting peer conflict and volume of self-reported antisocial behaviour at baseline.

The indirect path from delinquent peer association six months after randomisation to volume of self-reported antisocial behaviour 18 months after randomisation through beliefs and attitudes supporting peer conflict 12 months after randomisation was significant. Bias-corrected bootstrapped confidence intervals confirmed the statistical significance of the indirect effects (BC 95% CIs = 0.010–0.069). The total effect of delinquent peer association six months after randomisation on volume of self-reported antisocial behaviour 18 months after randomisation was not significant. The indirect effect accounted for 26% of the total effect of delinquent peer association 6 months after randomisation on volume of self-reported antisocial behaviour 18 months after randomisation. Fifteen percent of the variance in the volume of self-reported antisocial behaviour 18 months after randomisation was explained by the model.

Sensitivity testing revealed that unobserved covariates would need to correlate 0.25 with the mediator and dependent variable to eliminate the indirect effect of beliefs and attitudes supporting peer conflict on the peer influence effect observed. Findings were replicated when controlling for START treatment (MST or MAU) received, age and gender (indirect effect: *B* = 0.03, 95% CIs = 0.001–0.056), as well as when including peer conflict at 6 months and self-reported antisocial behaviour 12 months after randomisation as covariates (indirect effect: *B* = 0.01, 95% CIs = 0.001–0.033).

### Peer selection effect

**Fig. 2:**
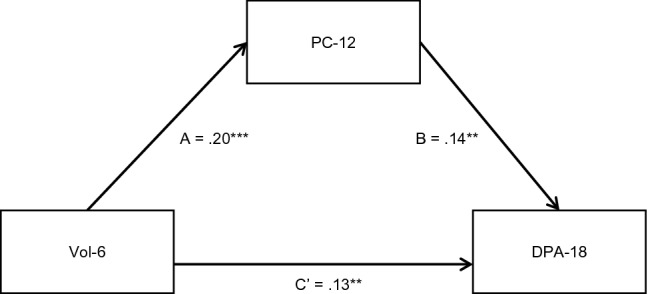
Path diagram for the peer selection mediation model. *N* = 673. Standardised beta coefficients are reported and precursor variables are not shown; **p < 01, ***p < 001, *Vol-6* volume of antisocial behaviour 6 months after randomisation, *PC-12* beliefs and attitudes supporting peer conflict 12 months after randomisation, *DPA-18* delinquent peer association 18 months after randomisation

Pairwise correlations between the variables specified in the peer selection model are presented in the supplementary materials Table C1. A mediation analysis was performed to examine whether beliefs and attitudes supporting peer conflict 12 months after randomisation mediated the relationship between volume of self-reported antisocial behaviour 6 months after randomisation and delinquent peer association 18 months after randomisation. Findings are presented in Table 3 and Fig. 2.

**Table 3:** Peer selection effect: direct, total and indirect effects

	Direct effects				
Path	*B* (95% CI)	*SE B*	β	*z*	*p*
Vol-6 to PC-12 (Path A)	0.05(0.031;0.067)	0.01	0.20	5.27	< 0.001
PC-12 to DPA-18 (Path B)	0.16(0.062;0.258)	0.05	0.14	3.20	0.001
Vol-6 to DPA-18 (Path C’)	0.04(0.013;0.064)	0.01	0.13	2.92	0.003
PC-baseline to PC-12	0.34(0.272;0.402)	0.03	0.38	10.13	< 0.001
DPA-baseline to DPA-18	0.14(0.062;0.212)	0.04	0.15	3.56	< 0.001

Total and indirect effects					
Vol-6 to DPA-18	*B* (95% CI)	*SE B*	β	*z*	*p*
Total Effect	0.05(0.021;0.072)	0.01	0.16	3.63	< 0.001
Indirect Effect	0.01(0.002;0.013)	0.00	0.03	2.70	0.007

*B (95% CI)* nstandardized coefficient with 95% confidence intervals, *SE B* standard error for the unstandardised beta coefficient, *β* standardised coefficient; *z* asymptotic z-test, *p* statistical significance level of the asymptotic z-test, *N* = 673, *PC-baseline* beliefs and attitudes supporting peer conflict at baseline, *PC-12* beliefs and attitudes supporting peer conflict 12 months after randomisation, *DPA-baseline* delinquent peer association at baseline, *DPA-18* delinquent peer association 18 months after randomisation, *Vol-6* volume of antisocial behaviour 6 months after randomisation

Volume of self-reported antisocial behaviour 6 months after randomisation had a significant direct effect on beliefs and attitudes supporting peer conflict 12 months after randomisation (Path A) and delinquent peer association 18 months after randomisation (Path C’), when controlling for the effect of beliefs and attitudes supporting peer conflict and delinquent peer association at baseline. Beliefs and attitudes supporting peer conflict 12 months after randomisation had a significant direct effect on delinquent peer association 18 months after randomisation (Path B), when controlling for the effect of beliefs and attitudes supporting peer conflict and volume of self-reported antisocial behaviour at baseline.

The indirect path from volume of self-reported antisocial behaviour 6 months after randomisation to delinquent peer association 18 months after randomisation through beliefs and attitudes supporting peer conflict 12 months after randomisation was significant. Bias-corrected bootstrapped confidence intervals confirmed the statistical significance of the indirect effects (BC 95% CIs = 0.003–0.015). The total effect of volume of self-reported antisocial behaviour 6 months after randomisation on delinquent peer association 18 months after randomisation was also significant. The indirect effect accounted for 17% of the total effect of volume of self-reported antisocial behaviour 6 months after randomisation on delinquent peer association 18 months after randomisation. Eight percent of the variance in the delinquent peer association scores 18 months after randomisation was explained by the model.

Sensitivity testing revealed that unobserved covariates would need to correlate 0.20 with the mediator and dependent variable to eliminate the indirect effect of beliefs and attitudes supporting peer conflict on the peer selection effect observed. Findings were replicated when controlling for START treatment (MST or MAU) received, age and gender (indirect effect: B = 0.01, 95% CIs = 0.002–0.014), as well as when including peer conflict at 6 months and delinquent peer association 12 months after randomisation as covariates (indirect effect: B = 0.01, 95% CIs = 0.003–0.027).

## Discussion

This study built upon past research investigating the role of certain forms of antisocial cognition as a mediator of the peer influence and peer selection effects. As expected, longitudinal analyses provided evidence in favour of both the peer selection and peer influence effects. Furthermore, we found evidence that beliefs and attitudes supporting peer conflict partially mediated the peer influence and peer selection effects, when controlling for prior levels of the mediator and dependent variables. The mediating effect of beliefs and attitudes explained 26 and 17% of the total effect in the peer influence and peer selection models, respectively. Sensitivity testing revealed that unobserved covariates would need to correlate 0.25 and 0.20 with the mediator and dependent variable to eliminate the indirect effects observed in the peer influence and peer selection models respectively. These correlations revealed in sensitivity testing are similar, if not higher, than previous research exploring the peer influence and peer selection effects [13, 14], and at levels suggesting modest robustness of the indirect effects to unobserved covariates [40].

These longitudinal findings are concordant with previous studies examining the associations between antisocial cognitions and peer influence and peer selection effects [12–15]. In addition, our study extends the external validity of previous research by investigating a novel mediator of the peer influence and peer selection effects. This novel mediator, namely antisocial beliefs and attitudes supportive of peer conflict, was rigorously measured in a large, mixed gender sample of adolescents with a history of serious antisocial behaviour. Our findings are also consistent with studies documenting a reciprocal relationship between the peer influence and peer selection effects [5–9]. In short, this was the first study to show that beliefs and attitudes supporting peer conflict may be involved in the transmission of the peer influence and peer selection effects, while highlighting that these antisocial cognitions may be a risk factor for delinquent peer association and vice-versa.

The developmental importance of antisocial beliefs and attitudes as a common possible mechanism for peer influence and peer selection effects is important to underline. One of the few studies that document the progression of antisocial beliefs and attitudes during adolescence found linear increases in attitudes tolerant of theft and violence and their behavioural counterparts across youth aged 6–18 years of age [41]. The mutual predictive power of attitude/behaviour relations was strongest in mid-to-late adolescence, congruent with the participants in our longitudinal study. More broadly speaking, the well-known cognitive and social advancements of adolescents suggest neuro-maturational changes that highlight both young people’s increasing capacity for reasoning and their sensitivity to peer networks [42]. Consequently, it is critical to understand young people’s developing beliefs and attitudes in relation to their peer ecologies.

These findings are also important in specifying a distinct domain of antisocial beliefs and attitudes as operative in the relationships between deviant peer associations and antisocial behaviour, rather than more general forms of antisocial cognition that are typically investigated in the youth offending literature, such as tolerance of law violations [43] or moral disengagement [25]. The significance of antisocial beliefs and attitudes supportive of peer conflict is congruent with fundamental insights gained from research into the developmental contributions and life-course trajectories of youth antisocial behaviour. Developmental scholars have identified that childhood aggression is a risk factor for early-onset and persistent antisocial behaviour [44, 45], which is promoted by deviant peer associations [46] and antisocial beliefs and attitudes during adolescence [47]. More recently, meta-analytic results suggest that antisocial beliefs and attitudes toward peer conflict, as they are operationalized “on the street” in peer networks, are related to youth offending and appear to be an important component of peer ecologies that promote youth criminogenic behaviour in urban communities [31]. Taken together, this literature suggests that the impact of antisocial beliefs and attitudes on youth offending follows developmental trajectories and is embedded in high-risk peer ecologies. Our results suggest it may be beneficial to complement the longstanding attention paid to the dynamics of parent–child interactions in the development of childhood aggressive and rule-breaking behaviour [48], with further research into the cognitive processes that support peer conflict in the development of serious and persistent antisocial behaviour during adolescence.

In terms of the peer influence effect, antisocial youth who associated with other deviant peers were more likely to endorse beliefs supportive of peer conflict, which, in turn, were associated with higher levels of antisocial behaviour at the final follow-up point. These findings suggest that deviant peer associations may increase the likelihood of engaging in further antisocial behaviour through shared beliefs and attitudes promotive of aggressive social relating and behaviour. These findings may point to an important role for antisocial beliefs and attitudes supportive of peer conflict in contributing to antisocial behaviour as a consequence of deviant peer influences.

Regarding the peer selection effect, levels of antisocial behaviour influenced the development of young people’s antisocial beliefs and attitudes supportive of peer conflict, suggesting that young people who were already engaging in relatively higher levels of antisocial behaviour, were more likely to endorse beliefs and attitudes supportive of peer conflict. These findings may point to an important role for antisocial beliefs and attitudes supportive of peer conflict in contributing to their deviant associations, which may then promote antisocial and offending behaviour.

The current findings support most major theories of offending to the extent that they identify antisocial cognition as a risk factor or consequence of delinquent peer association and antisocial behaviour [16, 32]. More specifically, they support the prediction from differential association theory [17, 18] that aspects of antisocial cognition, like beliefs and attitudes supporting peer conflict, constitute important mechanisms underpinning the peer influence effect. Moreover, contrary to social bond theory [32] and the General Theory of Crime [49, 50], they imply that antisocial beliefs and attitudes are involved in the transmission of the peer selection effect. Additionally, the findings are consistent with Interactional Theory [33], which specifies a dynamic, reciprocal relationship between delinquent peer association, antisocial cognition and antisocial behaviour through late childhood and early adolescence. Lastly, these findings may be applicable to social-cognitive information processing models of aggressive and antisocial behaviour. Integrated social–cognitive information-processing models elaborate two distinct but interacting domains that are central to the development and maintenance of antisocial acts in youth [51, 52], namely, “off-line” latent cognitive structures such as beliefs, attitudes, and values that endorse antisocial behavior and “on-line” cognitive decision-making processes, whereby a series of mental operations occur “in the moment” and within a specific context, such as making a biased hostile attribution [53], which leads young people to respond in an antisocial way. It is possible that the tendency to continue making hostile attributions “in the moment” is maintained, in part, by offline antisocial beliefs and attitudes supportive of peer conflict. This hypothesis would suggest that targeting antisocial beliefs and attitudes about peer conflict would help reduce the probability that young people would continue to engage in hostile attributions “in the moment” when confronted with ambiguous peer behaviour that could be interpreted in aggressive ways.

The results of this study may also have relevance for the concepts of *peer contagion* and *deviancy training*. Peer contagion refers to the mutual influence that occurs between peers and includes behaviours and emotions that can undermine individual development or cause harm to others [54]. The concept has been used to partly explain why some deviancy prevention programmes that place antisocial youth together appear to result in increased antisocial behaviour [47]. Deviancy training takes place when communications between peers about antisocial topics fosters engagement in antisocial acts and has been identified as a process of peer contagion [55]. The current findings suggest that deviancy training may partly account for peer contagion through the transmission of beliefs and attitudes supporting peer conflict between peers. If true, interventions to reduce antisocial behaviour that place antisocial youth together might benefit from trying to understand the extent that adolescents hold beliefs and attitudes supporting peer conflict and making such cognitions a target for intervention.

The study had several limitations that should be considered when interpreting the findings. First, it is not possible to advance causal inferences based on the findings because the study employed a non-experimental design [56–58]. Second, the relatively wide age range of participants at study entry (13–17 years) means we cannot say as to when the peer influence and peer selection effects were most likely to occur on the development trajectory, which may be worth exploring in subsequent research with a sufficient sample size to estimate potential moderating effects. Third, every variable was measured by self-report, which could have inflated effect estimates by way of shared method variance. Fourth, this analysis explored the independent presence of peer selection and peer influence effects, but did not model the effects simultaneously. Further research is needed to understand: (1) the relative importance of beliefs and attitudes supporting peer conflict against other potential mediators of the peer influence and peer selection effects; (2) if the mediation effects observed in this study remain when tested simultaneously rather than independently; and (3) the changing nature of these effects over time.

In sum, the findings from the mediation analyses support the view that antisocial beliefs and attitudes supportive of peer conflict may be fostered and reinforced by delinquent peer associations and antisocial behaviour, and that beliefs and attitudes supporting peer conflict could explain part of the mechanism underlying the peer influence and peer selection effects in adolescents with a history of serious antisocial behaviour. That is, when antisocial adolescents develop peer associations, they are likely to share and promote common beliefs and attitudes that value aggressive behaviour and ways of relating, making antisocial behaviour more likely. Moreover, antisocial adolescents’ involvement in antisocial behaviour is likely to consolidate such antisocial beliefs and beliefs, making deviant peer association more likely. Speculatively, antisocial young people associate and influence each other in part because of shared beliefs and attitudes about peer conflict, and, given these beliefs and attitudes about peer conflict, are more likely to be attracted to other antisocial young people. Over time, the outcomes are continued antisocial behaviour and deviant peer associations.

## Conclusion

This study suggests that beliefs and attitudes supporting peer conflict could explain part of the mechanism underlying the peer influence and peer selection effects in adolescents with a history of serious antisocial behaviour. This distinct domain of antisocial beliefs and attitudes requires greater attention in research attempting to understand the development of serious and persistent antisocial behaviour in young people. Moreover, the extent that adolescents with serious antisocial behaviour hold beliefs and attitudes supporting peer conflict should be considered when formulating interventions to reduce or prevent antisocial behaviour, especially those that place youth with a high risk of antisocial behaviour together.

## Supplementary Information

Below is the link to the electronic supplementary material.

Supplementary file1 (PDF 146 KB)
