# Supplementary material for: Antisocial cognition as a mediator of the peer influence effect and peer selection effect in antisocial adolescents
Source: Eur Child Adolesc Psychiatry. 2020 Dec 16;31(1):177–87. doi: 10.1007/s00787-020-01695-1 (PMC8816781; doi:10.1007/s00787-020-01695-1)
Supplement: Supplementary file 1 — Supplementary file1 (PDF 146 KB) [file 787_2020_1695_MOESM1_ESM.pdf]

Supplementary online materials to:

Antisocial Cognition as a Mediator of the Peer Influence Effect and Peer Selection Effect in Antisocial Adolescents

Supplementary materials A. Mediation analysis.

Three effects are stated in mediation analysis: direct, total and indirect. A direct effect is the degree to which a change in an upstream variable influences a change in a downstream variable without going through any other variable. In Figure 1, the direct effect of X on M, M on Y, and X on Y are represented by the path coefficients A, B and C, respectively. The total effect is equal to the sum of C + AB and reflects the extent that a change in X influences a change in Y regardless of M. The indirect effect is the product of path coefficients A and B and reflects the extent that a change in X influences a change in Y by means of M. Finally, the coefficient of C' represents the residual direct effect of X on Y after accounting for M. A full mediation process is empirically confirmed when C' equals zero and the indirect AB path (i.e., from X to Y via M) is statistically significant.

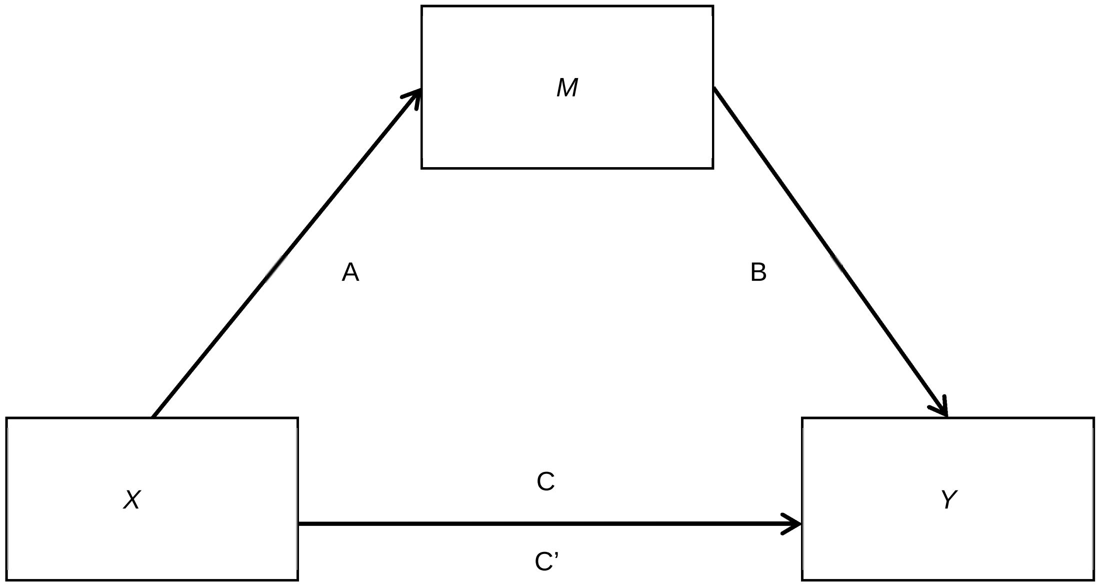

Figure 1. Conceptual path diagram for a simple mediation model.

In this study, criteria for mediation was a statistically significant indirect effect of the mediator (M) on the independent variable (X) - dependent variable (Y) relationship and a statistically non-significant or diminished direct effect of X on Y. As recommended by Wen and Fan (2015), the ratio of the indirect effect to the total effect was used as an effect size indicator for the indirect effect. The ratio of the indirect effect to the total effect is calculated by dividing the indirect effect by the total effect and is interpreted as the proportion of the total effect that is mediated [1, 2].

Supplementary materials B. Correlation analysis – Peer Influence effect.

Table B1 presents inter-correlations for the five variables specified in the peer influence mediation model and age, gender and treatment group (i.e., Multisystemic therapy and Management as usual).

Table B1: Inter-correlations for the five variables in the peer influence model and age, gender and treatment group

Variable	4	5	6	7	8
1. Age	.09	-.01	.03	.14	-.10
2. Gender	.07	.11	.06	.00	.02
3. Treatment	-.03	.01	-.01	.02	.03
4. PC-baseline		.46*	.13	.37*	.20*
5. PC-12			.15	.26*	.28*
6. DPA-6				.15	.17*
7. Vol-baseline					.37*
8. Vol-18					

Note. Age = age at study entry; Treatment = treatment group; PC-baseline = beliefs and attitudes supporting peer conflict at baseline; PC-12 = beliefs and attitudes supporting peer conflict 12 months after randomisation; DPA-6 = delinquent peer association six months after randomisation; Vol-baseline = volume of antisocial behaviour at baseline; Vol-18 = volume of antisocial behaviour 18 months after randomisation; * = statistically significant effect at $p < .002$ level (two tailed) (Bonferroni-corrected alpha: .05/25 significance tests). Pairwise deletion applied.

Eight of 25 correlations performed achieved statistical significance. There were small to medium positive correlations between independent, mediator and dependent variables and medium to large positive correlations between precursor variables and mediator/dependent variables. Age, gender and treatment group did not correlate with other variables and were therefore excluded from the peer influence mediation analysis. The relationship between delinquent peer association six months after randomisation and beliefs and attitudes supporting peer conflict 12 months after randomisation approached significance, $r(681) = .15$, $p = .004$.

Supplementary materials C. Correlation analysis – Peer Selection Effect

Table C1 presents inter-correlations for the five variables specified in the peer selection mediation model and age, gender and treatment group (i.e., Multisystemic therapy and Management as usual).

Table C1: Inter-correlations for the five variables in the peer selection model and age, gender and treatment group

Variable	4	5	6	7	8
1. Age	.09	-.01	.04	.02	0.11
2. Gender	.07	.11	.00	.01	-.02
3. Treatment	-.03	.01	-.01	-.01	.08
4. PC-baseline		.46*	.19*	.10	.30*
5. PC-12			.13	.20*	.33*
6. DPA-baseline				.21*	.12
7. DPA-18					.24*
8. Vol-6					

Note. Age = age at study entry; Treatment = treatment group; PC-baseline = beliefs and attitudes supporting peer conflict at baseline; PC-12 = beliefs and attitudes supporting peer conflict at 12 months after randomisation; DPA-baseline = delinquent peer association at baseline; DPA-18 = delinquent peer association at 18 months after randomisation; Vol-6 = volume of antisocial behaviour at six months after randomisation; * = statistically significant effect at $p < .002$ level (two tailed) (Bonferroni-corrected alpha: .05/25 significance tests). Pairwise deletion applied.

Seven of 25 correlations performed were statistically significant. There were small to medium positive correlations between precursor, independent, mediator and dependent variables. Age, gender and treatment group did not correlate with any other variables and were therefore excluded from the peer selection mediation analysis.

References

1. Preacher KJ, Kelley K (2011) Effect size measures for mediation models: Quantitative strategies for communicating indirect effects. Psychol Methods 16:93–115. https://doi.org/10.1037/a0022658
2. Wen Z, Fan X (2015) Monotonicity of effect sizes: Questioning kappa-squared as mediation effect size measure. Psychol Methods 20:193–203. https://doi.org/10.1037/met0000029
